# PD-L1^+^ Regulatory B Cells Are Significantly Decreased in Rheumatoid Arthritis Patients and Increase After Successful Treatment

**DOI:** 10.3389/fimmu.2018.02241

**Published:** 2018-10-01

**Authors:** Estefanía R. Zacca, Luisina I. Onofrio, Cristina D. V. Acosta, Paola V. Ferrero, Sergio M. Alonso, María C. Ramello, Eduardo Mussano, Laura Onetti, Isaac I. Cadile, Maria I. Stancich, Maria C. Taboada Bonfanti, Carolina L. Montes, Eva V. Acosta Rodríguez, Adriana Gruppi

**Affiliations:** ^1^Laboratorio de Inmunología, Hospital Nacional de Clínicas (HNC), Universidad Nacional de Córdoba (UNC), Córdoba, Argentina; ^2^Centro de Investigaciones en Bioquímica Clínica e Inmunología (CIBICI-CONICET), Facultad de Ciencias Químicas, UNC, Córdoba, Argentina; ^3^Servicio de Reumatología. Hospital Nacional de Clínicas, Universidad Nacional de Córdoba, Córdoba, Argentina

**Keywords:** rheumatoid arthritis, bregs, PD-L1, rheumatic diseases, inflammation

## Abstract

**Background:** B cells play an important role in the development and maintenance of rheumatoid arthritis (RA). Although IL-10–producing B cells represent a major subset of regulatory B cells (Bregs) able to suppress autoimmune and inflammatory responses, recent reports showed that B cell-mediated immune suppression may also occur independent of IL-10. For instance, B cells can modulate T cell immune responses through the expression of regulatory molecules such as PD-L1. So far, PD-L1-expressing B cells have not been analyzed in RA patients.

**Objective:** To analyze the frequency of PD-L1-expressing B cells in the peripheral blood of RA patients compared to healthy controls (HC) matched for sex and age, their function on T cell response and their changes in response to therapy.

**Methods:** Fresh peripheral blood B cells from RA patients and HC were characterized by flow cytometry and their functionality assessed in a co-culture system with autologous T cells.

**Results:** The frequencies of CD19^+^PD-L1^+^ B cells, CD24^hi^CD38^−^PD-L1^+^ and CD24^hi^CD38^hi^PD-L1^+^ B cells were significantly lower in untreated RA patients than in HC. In a follow-up study, the frequencies of PD-L1^+^ B cells (CD19^+^PD-L1^+^ B cells, CD24^hi^CD38^−^PD-L1^+^ and CD24^hi^CD38^hi^PD-L1^+^ B cells) increased significantly after treatment in good responder patients, although the frequency of total CD24^hi^CD38^hi^ B cells decreased. CD19^+^ B cells from untreated RA patients and HC upregulated PD-L1 expression similarly upon stimulation with CpG plus IL-2 and were able to suppress, *in vitro*, CD8^+^ T cell proliferation and cytokine production in a PD-L1-dependent manner.

**Conclusions:** Our results show that PD-L1^+^ B cells exhibiting T cell suppressive capacity are significantly decreased in untreated RA patients but increase in response to successful treatment. PD-L1 expression on B cells from RA patients can be modulated *in vitro* and PD-L1^+^ B cells could thus provide new perspectives for future treatment strategies.

## Introduction

Rheumatoid arthritis (RA) is a chronic, potentially debilitating inflammatory disease, characterized by destructive synovitis that, if left untreated, results in significant pain, swelling, stiffness, loss of function in the joints, deformity and disability (1). Tissue inflammation and damage is mediated through several cell types, including T cells, B cells, monocytes, macrophages, fibroblasts, and osteoclasts (2, 3). Clinical management goals in RA include enabling rapid access to optimum diagnosis and care. The treatment of RA has been revolutionized by the use of biologics, including TNF inhibitors, rituximab, abatacept, and others beyond standard therapy, generally methotrexate (MTX). One of the main pathways involved in RA, and also a target of treatment with abatacept, is the CD28-CD152 (CTLA-4) pathway (4, 5). In addition, inhibitory B7 family members, such as CD279 (PD-1) and CD274 (PD-L1), are also important in RA (6, 7). In mouse RA models, the deficiency of PD-1 or PD-L1 exacerbated the disease (8, 9). Thus, the PD-1/PD-L1 pathway appears as a potential immune checkpoint to control the inflammation in RA patients.

Regulation of the immune response is essential to maintain homeostasis and avoid autoimmunity. Recent advances in B cell biology have demonstrated that B cells, in addition to their capacity to produce antibodies, regulate innate, and adaptive immunity through the production of cytokines, such as IL-10, IL-35 and TGF-β (10, 11). RA patients exhibit a decrease in the percentage of IL-10-producing B cells (12, 13), and RA patients with active disease have fewer transitional CD19^+^CD24^hi^CD38^hi^ B cells, described as IL-10^+^ regulatory cells, in peripheral blood than patients with inactive disease or healthy individuals (14). Analysis of the changes in B cell subsets in RA patients, based exclusively on surface phenotype and not on functional activity, revealed that treatment with DMARDs leads to a further reduction in the absolute number of total B cells, switched memory and transitional B cells and plasmablasts (15).

B cells and plasmablasts can also modulate the T cell immune response through the expression of regulatory molecules, such as PD-L1 (16, 17). CpG induces PD-L1 expression on human B cells, which suppresses T helper type 2 cytokine production in pollen antigen-stimulated CD4-positive cells (18). Tumor-infiltrating IgA^+^ B cells that express high levels of PD-L1, IL-10 and TGF-β repress the proliferation and activation of CD8^+^ T cells (19). In addition, PD-L1^+^ B cells can suppress inflammation in experimental autoimmune encephalomyelitis (17). To evaluate whether changes in the frequency of regulatory B cell (Breg) populations are associated with clinical improvement of the disease, we analyzed the frequency of PD-L1-expressing peripheral B cell subsets in patients with RA under different treatments, and their functional activity on T cell response.

## Materials and methods

### Patients and healthy controls

Eighty consenting RA patients (age range, 22 to 83 years), under different treatment conditions or before receiving treatment (baseline, untreated), were recruited from the Rheumatology Service (Hospital Nacional de Clínicas, HNC). Sex- and age-matched consenting healthy controls (HC) were also recruited. RA patients were classified according to the American College of Rheumatology (ACR) and the European League against Rheumatism (EULAR) criteria (20). Exclusion criteria for RA patients included known or suspected ongoing infections, and neoplastic or inflammatory diseases. Exclusion criteria for HC included any history of autoimmune disease or immunosuppressive therapy. The clinical characteristics of the RA patients are summarized in Table 1. The RA disease activity score 28 (DAS28) was assessed at the time of blood collection, based on tender joint count, swollen joint count, visual analog scale score of the patient's global health and erythrocyte sedimentation rate (21). Response to treatment was defined according to EULAR criteria (22, 23). Good responder (R) and non-responder (NR) patients were classified using the individual change in the DAS28 value reached after treatment. A patient with a significant change (ΔDAS28 > 1.2) was classified as a R patient, and with a ΔDAS28 ≤ 0.6 as a NR patient. Moderate responders were not included in this study.

**Table 1:** Demographic and Clinical Features of RA Patients and Controls.

**Characteristic**	**RA patients**				**Healthy controls**
Age: range years	22–83				30–66
Sex: female/male	68/12				22/3
**Treatment:**	**Untreated**	**MTX**	**Anti-TNF**	**TOFA**	
CRP: median ± QD, mg/l	9.0 ± 7.5	5.0 ± 2.8	5.0 ± 3.9	5.5 ± 4.6	
ESR: mean ± SEM, mm/h	22 ± 3	13 ± 2	19 ± 4	20 ± 4	
DAS28 ESR: mean ± SEM	4.7 ± 0.3	3.3 ± 0.3	4.0 ± 0.3	3.6 ± 0.4	
Anti-CCP: positive/negative	17/9	12/8	14/6	10/4	
RF: positive/negative	19/7	11/9	14/6	11/3	

*CRP, C-reactive protein; ESR, erythrocyte sedimentation rate; DAS28, disease activity score 28; Anti-CCP, anti-citrullinated protein antibodies; RF, rheumatoid factor*.

Peripheral blood samples were collected from 25 HC, 26 untreated RA patients, 20 RA patients treated with methotrexate (MTX), 20 treated with TNF inhibitors (anti-TNF) and 14 treated with JAK inhibitor, Tofacitinib. In some patients, samples were obtained at baseline and after 3 months of treatment (*n* = 20). The study had the approval of the Hospital Nacional de Clínicas ethical committee (CIEIS) and was conducted according to the Declaration of Helsinki on studies with human subjects. A written informed consent was obtained from patients and controls prior to any study procedure.

### Erythrocyte sedimentation rate, C-reactive protein, rheumatoid factor and anti-citrullinated protein antibodies determination

Peripheral blood samples anticoagulated by EDTA-K2 or by sodium citrate 3.8% W/V were collected for cytological analysis and flow cytometry and for erythrocyte sedimentation rate determination, respectively. Serum samples were obtained for autoantibody determinations. Levels of CRP were determined by a particle-enhanced turbidimetric immunoassay (SIEMENS), using the autoanalyzer (SIEMENS Dimension RXL Max). Rheumatoid Factor **(**RF) was determined by a latex agglutination test (Artritest, Wiener Laboratories) and anti-citrullinated cyclic peptide antibodies were quantified by ELISA, according to the manufacturer's instructions (Orgentec Diagnostika Gmbh).

### Flow cytometry

For surface staining, 200 ul of anticoagulated peripheral blood were stained during 30 min at room temperature with a combination of the following Abs: anti-CD19 PerCPCy5.5 (HIB19, BD), anti-CD19 APCCy7 (HIB19, Biolegend), anti-CD24 FITC (ML5, BD), anti-CD38 APC (HIT2, BD), anti-PD-L1 PECy7 (MIH1, BD), control isotype (MOPC-21, BD), anti-CD4 FITC (13B8.2, Beckman Coulter, Brea, CA), anti-CD8 PerCP, and anti-CD8 APC (RPA-T8, eBioscience. After staining, red blood cells were lysed with 5 ml of cold lysing buffer (NH4Cl 0.15M, KHCO3 10 mM, Na2EDTA 0.1 mM, in distilled water) during 20 min at 4°C. Then, samples were centrifuged, washed with PBS and resuspended in 2%FBS-PBS and acquired on a BD FACSCanto II Flow Cytometry. The analysis was performed using FlowJo software (version X).

### Cell separation procedures

Fresh peripheral blood mononuclear cells (PBMCs) from heparinized blood samples obtained from HC and RA patients were isolated via centrifugation over a Ficoll-Hypaque gradient (GE Healthcare Bio-Science AB). Viability was determined by trypan blue exclusion. B cells were positively selected by anti-CD19-coated magnetic particles (EasySep Stem Cell), according to the manufacturer's instructions and were >98% pure as assessed by flow cytometry. CD8^+^ and CD4^+^ cells were isolated with a positive CD8^+^ and CD4^+^ T cell selection kit (EasySep Stem Cell) and resulted in >98% CD8^+^ cells and >97% CD4^+^ cells.

### Cell cultures

Freshly isolated B cells were cultured in complete medium [RPMI 1640 medium supplemented with 10% heat-inactivated fetal bovine serum (FBS), 100 units/ml of penicillin, 100 ug/ml of streptomycin, 1 mM L-glutamine, 10 mM HEPES (all from Gibco) and 2-mercaptoethanol (Sigma)] for 3 days in 96-well plates at 1 × 10^5^ B cells/well in the presence of recombinant human IL-2 (40 ng/ml; Biolegend) and CpG-ODN 2006 (1 ug/ml; Invivogen), at 37°C in a fully humidified atmosphere containing 5% CO_2_. For assessment of B cell IL-10 production, freshly isolated PBMC were also stimulated with 50 ng/ml phorbol 12-myristate 13-acetate (PMA, Sigma-Aldrich) and 1 μg/ml ionomycin (Sigma-Aldrich) for the last 15 h in the presence of Brefeldin A (GolgiPlug, BD; PIB). After stimulation, cells were washed and stained during 30 min at 4°C with anti-CD19 APCCy7 (HIB19, Biolegend) and anti-PD-L1 PECy7 (MIH1, BD). Subsequently, cells were washed, fixed, and permeabilized for 20 min at 4°C using Cytofix/Cytoperm (BD). Cells were washed twice with Perm/Wash (BD) and stained for 30 min at room temperature with anti-IL-10 Alexa Fluor 647 (JES3-9D7, Biolegend). Intracellular cytokine production was analyzed on CD19^+^ lived cells.

To evaluate T cell proliferation, purified CD4^+^ and CD8^+^ T cells (0.1 × 10^6^) were resuspended in PBS-FBS 1% and stained with 1 μM Carboxyfluorescein succinimidyl ester (CFSE). Then, cells were stimulated in 96-well plates (Costar, flat bottom) pre-coated overnight with anti-CD3 (0.5 μg/ml, OKT3, Biolegend) and anti-CD28 (0.25 μg/ml, CD28.2, Biolegend) in presence or absence of stimulated CD19^+^ B cells. In some experiments, anti-PD-L1 mAb (29E.2A3, Biolegend) or control isotype (MPC-11, Biolegend) were added to the wells. After 3 days of culture, cells were further stimulated with PMA/Ionomycin to determine cytokine production as described below. Proliferation was determined as CFSE dilution by flow cytometry. Fixable Viability Stain 780 at day 3 of culture was used for exclusion of dead cells. The proliferative response of T cells stimulated with anti-CD3/CD28 alone was considered as 100% and the proliferation in the presence of activated B cells with anti-PD-L1 or with isotype control mAb was calculated relative to that 100%.

For assessment of T cell cytokine production, the cell cultures were stimulated with PIB for 2 h. After stimulation, cells were washed and stained during 30 min at 4°C with anti-CD19 APCCy7 (HIB19, Biolegend) and anti-CD3 APC (UCHT-1, BD). Subsequently, cells were washed, fixed and permeabilized for 20 min at 4°C using Cytofix/Cytoperm (BD). Cells were washed twice with Perm/Wash (BD) and stained for 30 min at room temperature with anti-IFN-gamma (IFN-g) PerCP (B27, eBioscience) and anti-TNFα PECy7 (MAb11, BD). Intracellular cytokine production was analyzed on CD3^+^ CD19^−^ lived cells.

### Statistics

Statistical analyses were performed with GraphPad Prism version 7 software (GraphPad Software). *P*-values < 0.05 were considered significant. The D'Agostino-Pearson normality test was initially performed to determine the distribution of the datasets. For normally distributed data, statistical testing was performed with Student's paired *t*-test, Student's unpaired *t*-test, one-way ANOVA followed by the Bonferroni post-test and Pearson's correlation coefficient. For non-normally distributed data, statistical testing was performed with the Mann-Whitney test, the Kruskal-Wallis test with Dunn's correction and Spearman's correlation test.

## Results

### RA patients exhibited similar percentages of memory and mature B cells and plasmablasts but anti-TNF and tofacitinib-treated patients exhibited low percentages of CD24^hi^CD38^hi^ B cells

**Figure 1:**
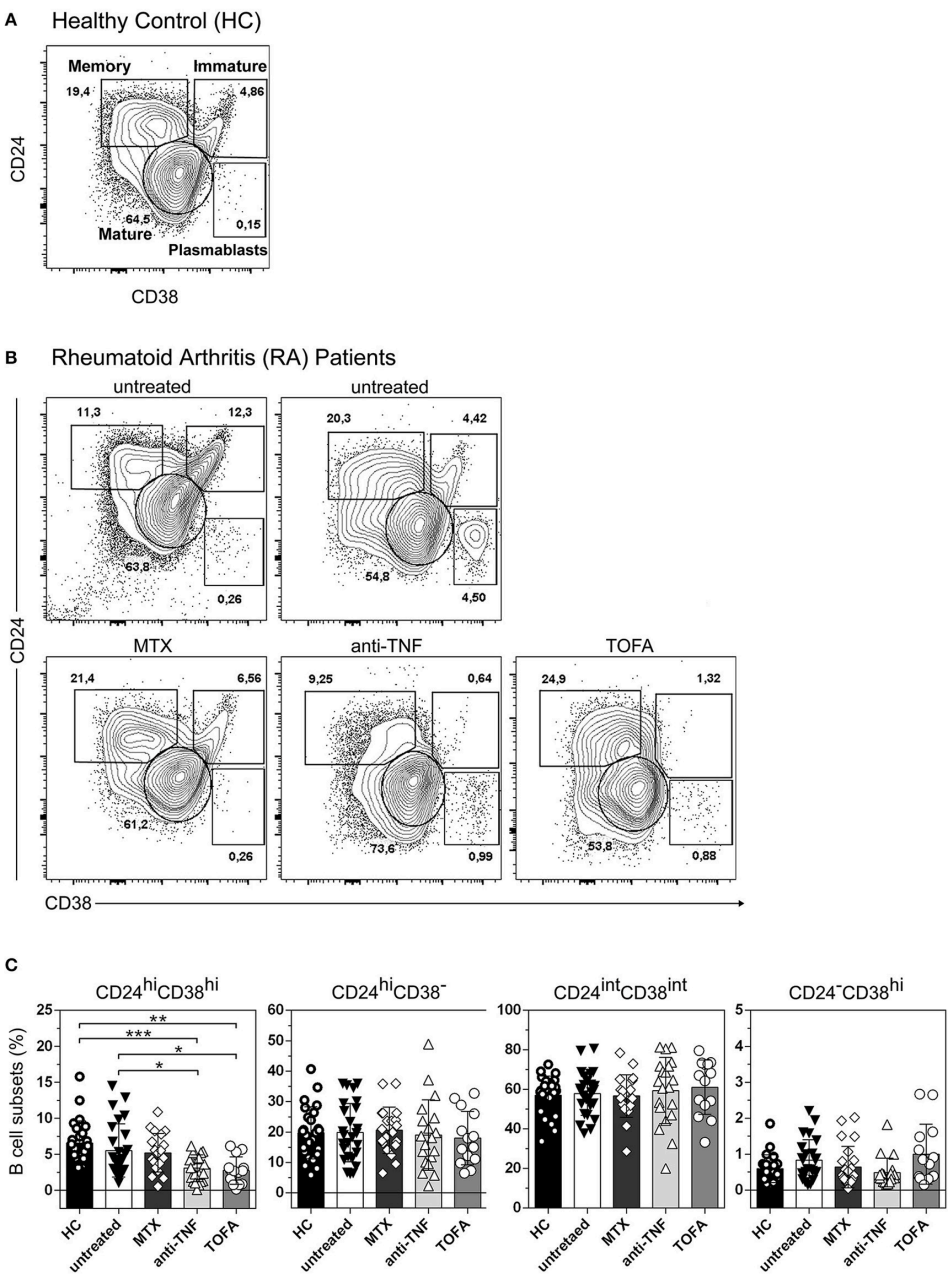
Changes in peripheral blood B cell subsets in RA patients following different treatments. Representative dot plot with gating strategies for B cell subsets: CD19^+^CD24^hi^CD38^hi^ B cells (immature B cells), CD19^+^CD24^int^CD38^int^ B cells (mature B cells), CD19^+^CD24^hi^CD38^−^ B cells (primarily memory B cells) and plasmablasts (CD19^+^CD24^−^CD38^hi^) from **(A)** healthy controls (HC) and **(B)** untreated RA patients or RA patients treated with methotrexate (MTX), anti-TNF or Tofacitinib (TOFA). Flow cytometry analysis was performed on CD19^+^ B cells. **(C)** Bar charts show frequencies of immature (CD24^hi^CD38^hi^), memory (CD24^hi^CD38^−^), mature (CD24^int^CD38^int^) and plasmablast (CD24^−^CD38^hi^) CD19^+^ B cells in HC (*n* = 25), untreated (*n* = 26) or treated RA patients (MTX, *n* = 20; anti-TNF, *n* = 20; TOFA, *n* = 14). Data are expressed as mean ± SEM. *p < 0.05, **p < 0.01, ***p < 0.001. Kruskal-Wallis test with Dunn's correction were used.

Four distinct B cell populations can be identified in human peripheral blood: CD19^+^CD24^hi^CD38^hi^ B cells (mainly immature B cells), CD19^+^CD24^int^CD38^int^ B cells (mature B cells), CD19^+^CD24^hi^CD38^−^ B cells (memory B cells) and CD19^+^CD24^−^CD38^hi^ (plasmablasts) (24–26) (Figure 1A). To evaluate changes in the frequency of B cell subsets in our cohort of patients, we first analyzed by flow cytometry the percentages of the different peripheral B cell populations in patients with RA at diagnosis and under different treatments, as well as in healthy individuals. For this purpose, peripheral blood samples were obtained from healthy controls (HC, *n* = 25), untreated RA patients (untreated, *n* = 26) and RA patients treated with methotrexate (MTX, *n* = 20), TNF inhibitors (anti-TNF, *n* = 20), and the JAK inhibitor Tofacitinib (TOFA, *n* = 14). The gate strategy used to define B cell subsets is illustrated by the representative staining in cells from HC subjects (Figure 1A) and from RA patients under different treatment conditions (Figure 1B). Figure 1C shows that the frequencies of memory (CD24^hi^CD38^−^), mature (CD24^int^CD38^int^) B cells and plasmablasts (CD24^−^CD38^hi^) were not significantly different between RA patients and HC. However, a significant decrease in the frequency of immature CD24^hi^CD38^hi^ B cells was observed in RA patients treated with anti-TNF or Tofacitinib compared to untreated RA patients and HC. Considering that the CD24^hi^CD38^hi^ B cell subset (27, 28) was assigned regulatory functions, we asked whether the decrease in CD24^hi^CD38^hi^ B cells in RA patients treated with anti-TNF and with Tofacitinib was related to the status of the disease. In these patients we found a tendency, although without statistical significance (*r* = −0.3; *p* = 0.1454), to negative correlation between the frequency of CD24^hi^CD38^hi^ B cells and disease activity, determined as DAS28 (data not shown). This supports previous reports (29–31) that the reduction of a B cell subset with regulatory properties assigned may be associated with high autoreactive response and inflammation.

### The frequency of CD24^hi^CD38^−^ B cells in untreated RA patients correlated negatively with DAS28

**Figure 2:**
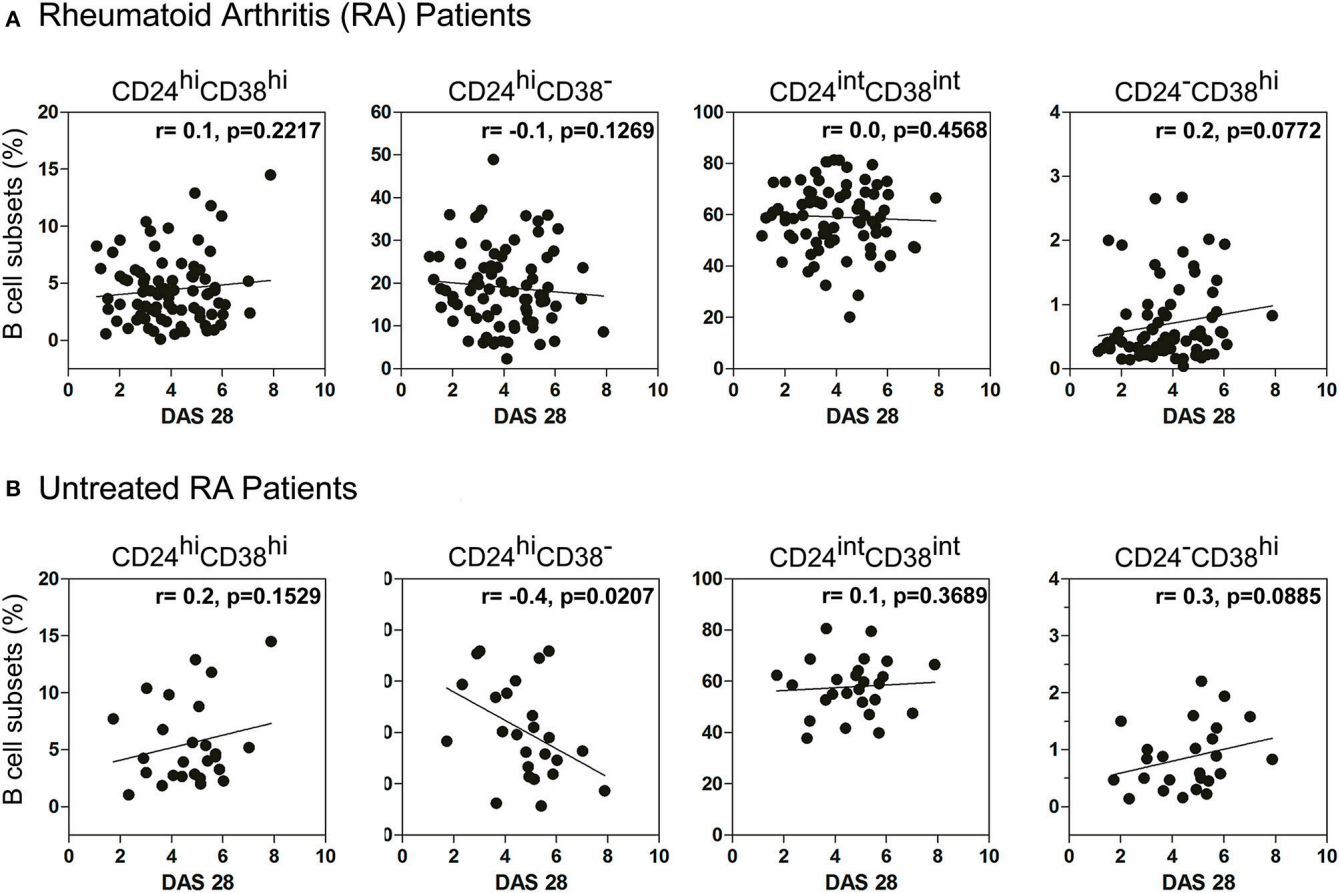
Correlation between DAS28 and the frequency of B cell subsets. Correlation plots show the relationship between DAS28 and the frequencies of immature (CD24^hi^CD38^hi^), memory (CD24^hi^CD38^−^), mature (CD24^int^CD38^int^) and plasmablast (CD24^−^CD38^hi^) CD19^+^ B cells in **(A)** treated and untreated RA patients (*n* = 80) and **(B)** untreated RA patients (*n* = 26). Correlation analysis was performed with Pearson's correlation test and a p value < 0.05 was considered statistically significant.

Considering that RA patients exhibit different inflammation status and disease progression, we next decided to examine a possible association between the frequency of B cell subsets and DAS28 in RA patients. No significant correlation was observed between the percentages of immature, memory, mature B cells, and plasmablasts with DAS28 (Figure 2A) in all the RA patients enrolled in the study. However, interestingly, the frequency of peripheral CD24^hi^CD38^−^ B cells correlated negatively with disease activity in patients who did not receive any specific treatment (*r* = −0.4; *p* = 0.0207; Figure 2B), suggesting that, in untreated RA patients, a better status is associated with a greater frequency of these cells.

### Frequency of CD24^hi^CD38^−^ B cells increased while CD24^hi^CD38^hi^ B cells decreased in good responder patients

**Figure 3:**
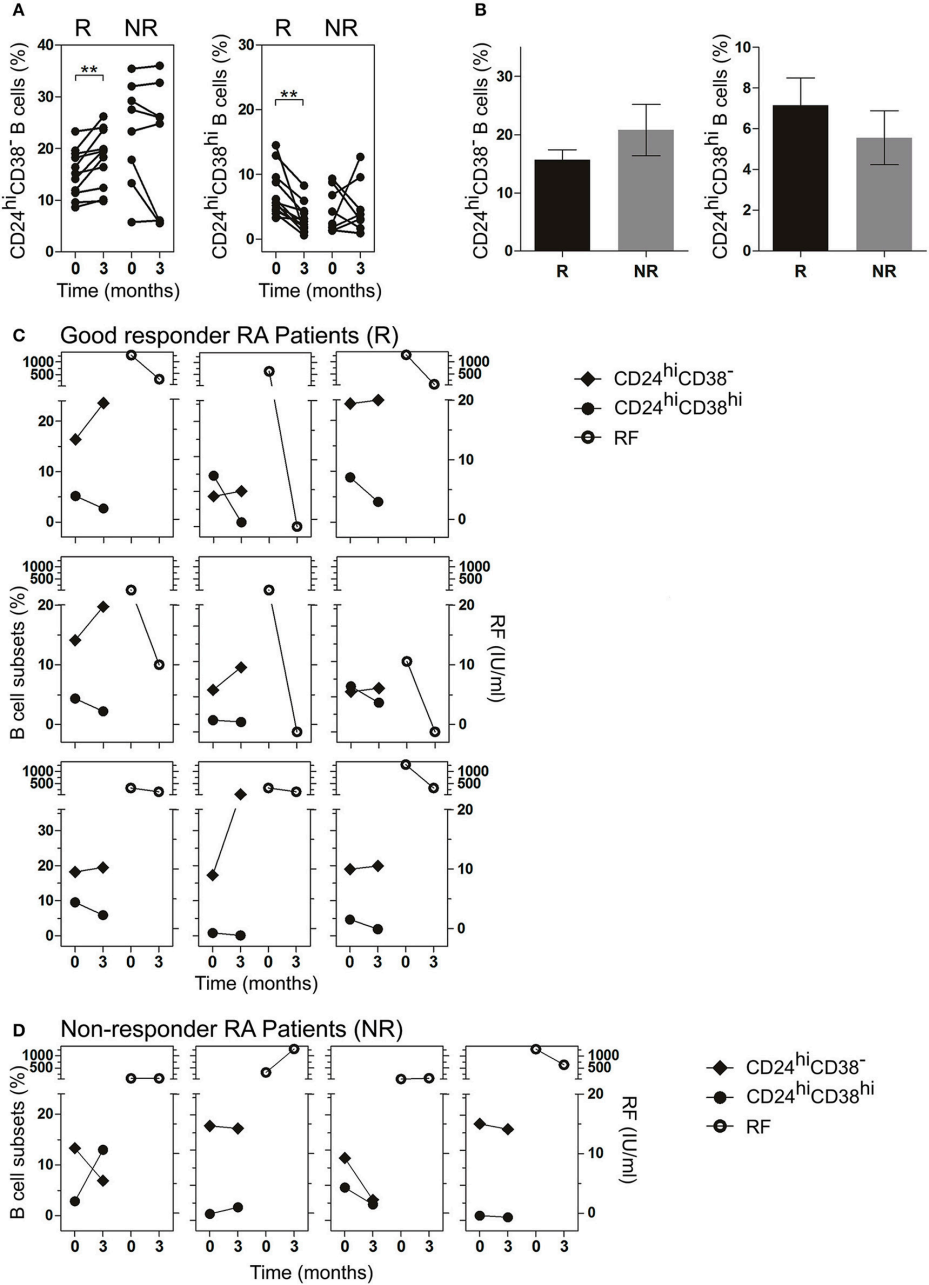
Frequency of CD24^hi^CD38^−^ and CD24^hi^CD38^hi^ B cells in peripheral blood of RA patients at baseline and after treatment. **(A,B)** Frequency of CD24^hi^CD38^−^ and CD24^hi^CD38^hi^ B cells at baseline (time 0) and after 3 months (time 3) of different treatments **(A)** or at baseline (time 0) depicted in bars **(B)** in good responder (R, *n* = 12) and non-responder (NR, *n* = 8) patients. **p < 0.01. P values were determined using the Student's *t*-test for paired samples. **(C,D)** Representative graphs showing the frequency of B cell subsets (left) and rheumatoid factor levels (right) at baseline (time 0) and after 3 months (time 3) of treatment in R patients (*n* = 9; **C)** and NR patients (*n* = 4; **D)** seropositive for RF.

RA patients had different responses to drug therapy and were classified as good, moderate or bad responders to treatment, according to EULAR criteria (22, 23). To analyze the changes in B cell subsets in response to treatment, each patient was studied individually in a follow-up study according to the clinical response (R: good responder and NR: non-responder) after 3 months of treatment (as indicated in Material and Methods). After 3 months of treatment, R patients exhibited a significant increase in the frequency of CD24^hi^CD38^−^ B cells and a significant decrease in CD24^hi^CD38^hi^ B cells (Figure 3A). Random changes were observed in the frequencies of both populations in NR RA patients. The frequency of CD24^hi^CD38^−^ and CD24^hi^CD38^hi^ B cells in both R and NR patients before receiving treatment was analyzed and non-significant differences were observed in the baseline data between both groups (Figure 3B).

Interestingly, in R patients seropositive for RF, lower frequencies of CD24^hi^CD38^hi^ B cells and higher frequencies of CD24^hi^CD38^−^ B cells after treatment were associated with decreased RF levels (Figure 3C). This association was not observed in NR RA patients (Figure 3D), in which the changes in the levels of RF and the percentages of B cell subsets did not follow a common pattern.

### CD24^hi^CD38^−^ and CD24^hi^CD38^hi^ B cells that expressed PD-L1 were significantly decreased in untreated RA patients

**Figure 4:**
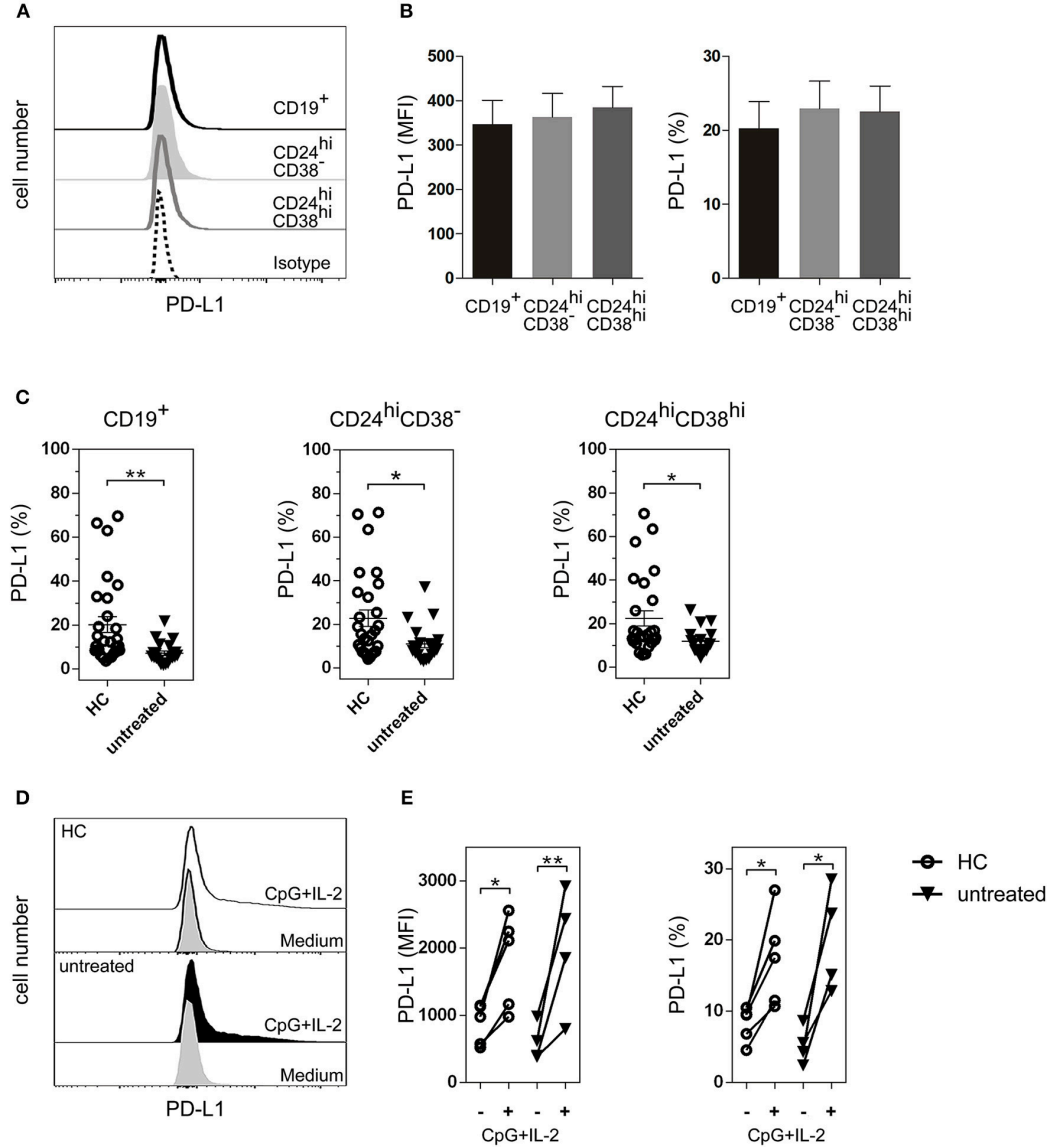
PD-L1^+^ B cell subsets in HC and untreated RA patients. **(A)** Representative histogram of PD-L1 expression on total CD19^+^ B cells and on CD24^hi^CD38^−^ and CD024^hi^CD38^hi^ B cell subsets from HC. **(B)** Expression (mean fluorescence intensity (MFI) left panel and frequency, right panel) of PD-L1 on B cell subsets from peripheral blood of HC (*n* = 25). Data are expressed as mean ± SEM. Kruskal-Wallis test with Dunn's correction were used. **(C)** Frequency of CD19^+^PD-L1^+^ B cells, CD24^hi^CD38^−^PD-L1^+^ and CD24^hi^CD38^hi^PD-L1^+^ B cell subsets from HC (*n* = 25) and untreated RA patients (*n* = 24). Data are expressed as mean ± SEM. **p < 0.01. P values were determined using the Mann-Whitney Test. (**D)** Representative histograms of PD-L1 expression on total CD19^+^ B cells purified from PBMC of HC and untreated RA patient after 72 h of incubation with CpG+IL-2 or with medium alone. **(E)** Expression (MFI, left panel and frequency, right panel) of CD19^+^PD-L1^+^ B cells purified from PBMC of HC (*n* = 5) and untreated RA patients (*n* = 4) after 72 h of incubation with CpG+IL-2 or with medium alone. Data were collected from five independent experiments. *p < 0.05, **p < 0.01. P values were determined using the Student's *t*-test for paired samples.

Although the majority of Bregs described in mice and humans have been identified by IL-10 production (32), cells of the B lineage can also regulate immunity through PD-L1 (17, 33). PD-L1 functions as a regulatory protein to maintain T cell self-tolerance (34, 35), and may play a role in Breg activity in RA. Therefore, we next studied PD-L1 expression on B cell subsets. As shown in Figure 4A, PD-L1 was expressed in total CD19^+^ B cells as well as in CD24^hi^CD38^−^ and CD24^hi^CD38^hi^ B cell subsets. The levels of PD-L1 expression, defined by the mean of fluorescence intensity (MFI) and the frequency of PD-L1^+^ cells, were similar in all the analyzed B cell populations studied *ex vivo* in whole peripheral blood (Figure 4B). Notably, untreated RA patients had a significant decrease in the frequencies of CD19^+^PD-L1^+^ as well as in CD24^hi^CD38^−^PD-L1^+^ and CD24^hi^CD38^hi^PD-L1^+^ B cells compared to HC (Figure 4C). Interestingly, most of the PD-L1^+^ B cells were not IL-10-producing cells (Figure S1).

### CD19^+^ B cells incubated with CPG+IL-2 increased PD-L1 expression

Next, we asked whether the decrease in the frequency of PD-L1^+^ B cells in RA patients was due to an intrinsic defect of the B cells from these patients to express this molecule, or whether these B cells could upregulate PD-L1 under certain stimuli. PD-L1 increases when purified human B cells are incubated with CpG nucleotides (18, 36). To determine the ability of CD19^+^ B cells from untreated RA patients to upregulate PD-L1, we purified CD19^+^ B cells from PBMC of untreated RA patients and incubated them during 72 h with medium alone or with CpG+IL-2. The addition of exogenous IL-2 provides optimal B cell survival condition. CD19^+^ B cells from HC were used as a control. B cells from both untreated RA patients and HC were equally able to upregulate PD-L1 expression, as shown in the representative histograms of CD19^+^-gated B cells (Figure 4D). Both the MFI of PD-L1 expression and the frequency of CD19^+^PD-L1^+^ B cells increased significantly in HC and untreated RA patients upon stimulation (Figure 4E), suggesting that B cells from RA patients can upregulate this inhibitory molecule under appropriate conditions. Neither medium nor IL-2 alone increased the expression of PD-L1 on CD19^+^ B cells (Figure S2).

### CD19^+^ B cells incubated with CPG+IL-2 suppressed T cell response via PD-L1

**Figure 5:**
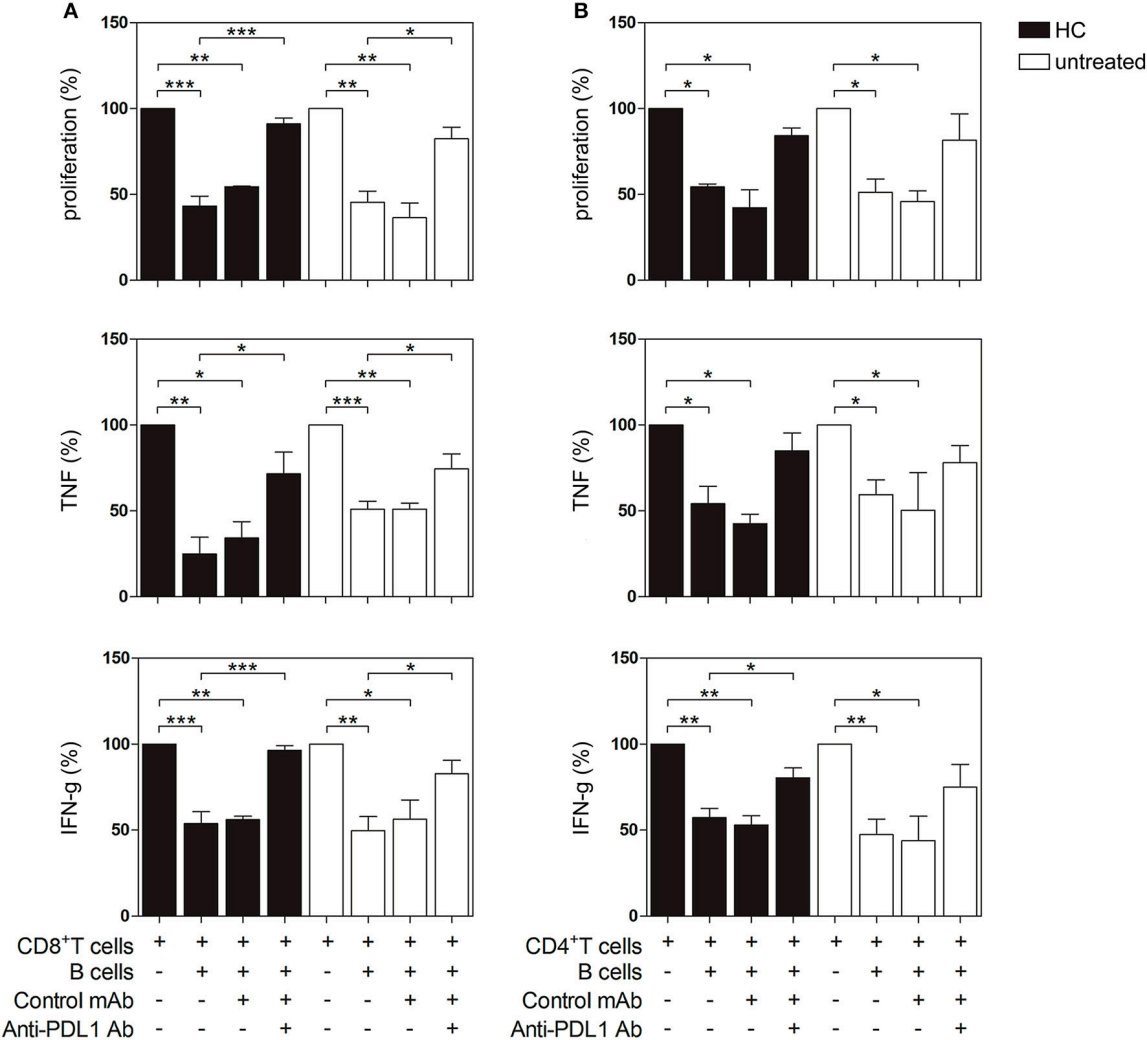
PD-L1-dependent suppression of T cell proliferation and cytokine production by PD-L1 expressing B cells from HC and untreated RA patients. CD19^+^ B cells, CD8^+^ and CD4^+^ T cells were magnetic bead-purified from PBMC of HC and untreated RA patients. CD19^+^ B cells were incubated with CpG+IL-2 for 72 h and then were washed and cultured 2:1 with autologous CFSE-labeled CD8^+^ or CD4^+^ T cells incubated in a plate-bound anti-CD3/anti-CD28 mAb in the presence of anti-PD-L1 mAb or control isotype. After 72 h of co-culture, T cell proliferation and intracellular cytokine production were analyzed by flow cytometry on CD3^+^ CD19^−^ lived cells. Percentage of proliferation, TNF and IFN-g production in **(A)** CD8^+^ T cells and **(B)** CD4^+^ T cells cultured alone or in the presence of CpG-activated CD19^+^ B cells with anti-PD-L1 mAb or control isotype. Mean ± SEM of five independent experiments (5 healthy controls and 5 untreated RA patients). *p < 0.05, **p < 0.01, ***p < 0.001. One-way ANOVA followed by a Bonferroni's post-test were used.

Next, we investigated the regulatory capacity of PD-L1^+^ B cells on CD4^+^ and CD8^+^ T cell proliferation and cytokine production. Briefly, B cells were incubated with autologous previously CD3/CD28-stimulated T cells. Unstimulated B cells were not able to suppress the production of TNF by CD8^+^ T cells (Figure S3). However, as shown in Figures 5A,B, when CpG-stimulated CD19^+^ B cells from HC (black bars) were added to the T cell culture, the proliferation of CD8^+^ and CD4^+^ T cells and the frequency of TNF^+^ and IFN-g^+^ CD8^+^ and CD4^+^ T cells significantly decreased. Notably, CD19^+^ B cells from untreated RA patients were also able to suppress CD8^+^ and CD4^+^ T cell proliferation and intracellular cytokine production (white bars). To explore the role of PD-L1 in our co-culture systems, we performed blocking experiments with an anti-PD-L1 mAb. Figure 5A shows that blockade of PD-L1 significantly increased CD8^+^ T cell proliferation as well as the frequency of cytokine-producing CD8^+^ T cells from both HC and untreated RA patients, while an isotype control mAb did not modify B cell suppressor activity. Figure 5B shows that blockade of PD-L1 significantly reverted the frequency only of IFN-g^+^ CD4^+^ T cells from HC but not of their counterparts from RA patients. It should be noted that, although there was a tendency of anti-PD-L1 to revert the suppression of CD4^+^ T cells triggered by CpG-stimulated B cells, it was not significant, suggesting that B cells from HC and RA patients may suppress a CD4^+^ T cell response not exclusively via PD-L1 but using other mechanisms/mediators.

### PD-L1^+^ B cells were increased after treatment in good responder patients

**Figure 6:**
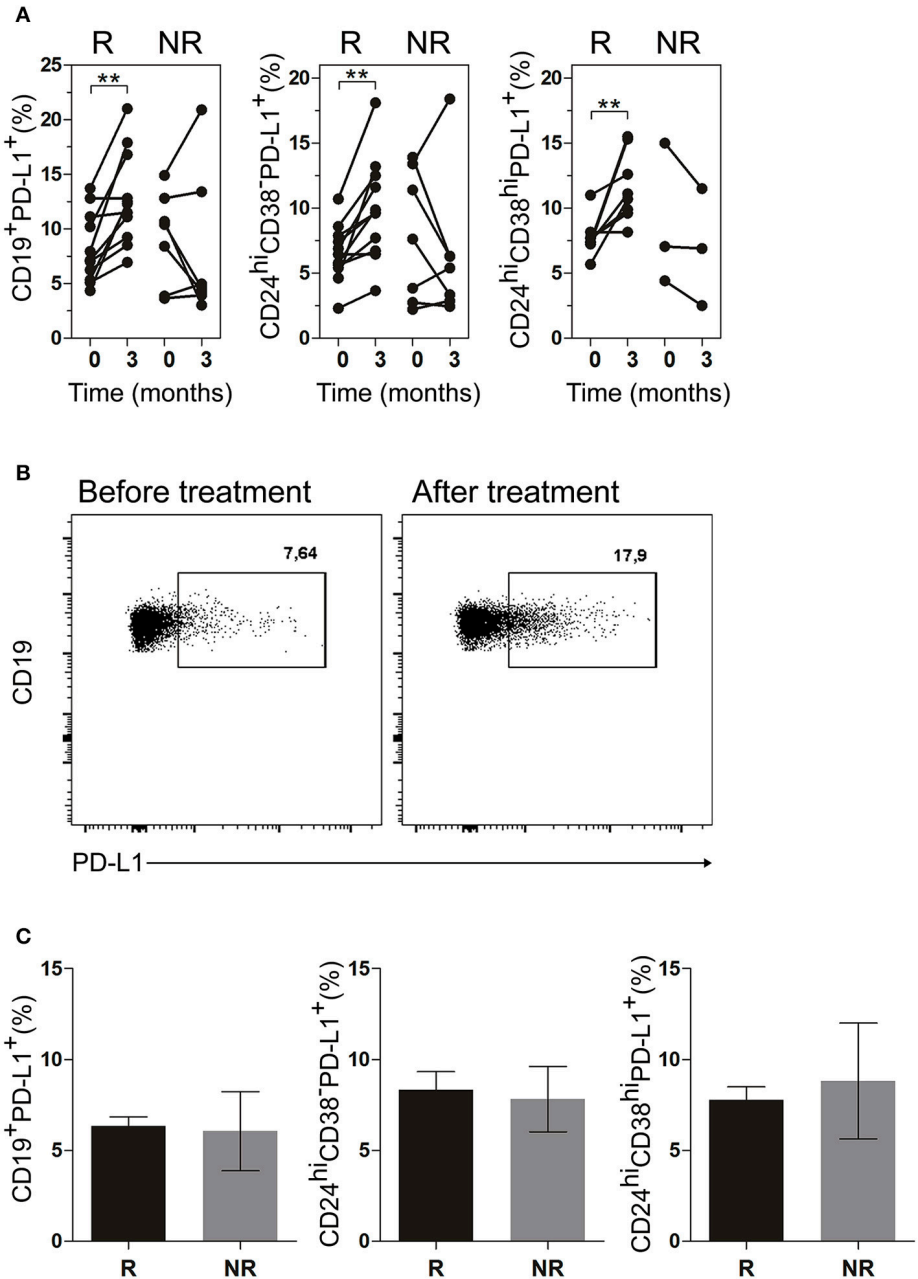
CD19^+^PD-L1^+^, CD24^hi^CD38^−^PD-L1^+^ and CD24^hi^CD38^hi^ PD-L1^+^ B cells were increased in peripheral blood of good responder patients. **(A)** Frequencies of CD19^+^PD-L1^+^ B cells, CD24^hi^CD38^−^PD-L1^+^ and CD24^hi^CD38^hi^PD-L1^+^ B cells at baseline (time 0) and after 3 months (time 3) of different treatments in good responders (R, *n* = 11) and non-responders (NR, *n* = 7) patients. **(B)** Representative dot plot of PD-L1^+^ vs CD19^+^ B cells in a R patient, at baseline and after 3 months of treatment. Flow cytometry analysis was performed on lymphoid cells. **p < 0.01. P values were determined using the Student's *t*-test for paired samples. **(C)** Frequencies of CD19^+^PD-L1^+^ B cells, CD24^hi^CD38^−^PD-L1^+^ and CD24^hi^CD38^hi^PD-L1^+^ B cells at baseline (time 0) in good responder (R, *n* = 11) and non-responder (NR, *n* = 7) patients. Data are expressed as mean ± SEM. Student's unpaired *t*-test was used.

Next, we evaluated the frequency of PD-L1^+^ B cells in the patients undergoing treatment. Figure 6A illustrates a significant increase in R patients in the frequencies of CD19^+^PD-L1^+^ B cells as well as of CD24^hi^CD38^−^PD-L1^+^ and CD24^hi^CD38^hi^PD-L1^+^ B cell subsets after 3 months of treatment. In contrast, NR patients exhibited random changes in the frequencies of CD19^+^PD-L1^+^ B cells, CD24^hi^CD38^−^PD-L1^+^ and CD24^hi^CD38^hi^PD-L1^+^ B cells. Given the very low percentages of CD24^hi^CD38^hi^ B cells in NR patients, we were able to accurately determine the frequency of CD24^hi^CD38^hi^PD-L1^+^ B cells in just three NR patient (Figure 6A, right panel). Figure 6B shows a representative dot plot of the frequency of CD24^hi^CD38^−^PD-L1^+^ B cells at baseline and after 3 months of treatment in a representative R patient. The frequency of CD19^+^PD-L1^+^, CD24^hi^CD38^−^PD-L1^+^, and CD24^hi^CD38^hi^PD-L1^+^ B cell in both R and NR patients before receiving treatment was analyzed and non-significant differences were observed in the baseline data between both groups (Figure 6C).

## Discussion

In order to predict the response of patients to a particular therapy there is a need to identify markers that will recognize which patients will respond to treatment. For this purpose, the frequency of B lineage cells with regulatory function (37, 38) and Tregs have been proposed as potential biomarkers (39, 40). We studied the frequency of CD19^+^ PD-L1^+^ cells in both R and NR patients before receiving treatment and we have not observed significant differences in the baseline data that allow us to predict a differential response. However, an increase in the frequency of these cells, with *in vitro* regulatory function, was associated with a positive response to treatment.

The role of B cell subsets with regulatory functions in autoimmune diseases was previously reported. It has been shown that human CD19^+^CD24^hi^CD38^hi^ B cells possessed regulatory capacity and that patients with Systemic Lupus Erythematosus (SLE) (27) or with inactive RA (14) had similar peripheral blood levels of CD19^+^CD24^hi^CD38^hi^ B cells to those of healthy individuals. However, RA patients with active disease have reduced numbers and regulatory activity of these CD19^+^CD24^hi^CD38^hi^ B cells (14). Salomon et al. (38) also found that CD24^hi^CD38^hi^ B cells were significantly reduced in RA patients at baseline compared with controls. Iwata et al. identified a subset of human regulatory B cells with a phenotype of CD24^hi^CD27^+^, approximately 60% of which express CD38 (41) and are significantly higher in patients with autoimmune disease than in HC. We found no significant differences in the frequency of mature, memory, and plasmablast B cell subsets in our cohort of RA patients with respect to HC when we analyzed all the patients enrolled in the study, treated and untreated, without considering the status of the disease or response to treatment. Our results match those of Daien et al. (12), who reported that CD24^hi^CD38^hi^, CD24^hi^CD27^+^ and CD5^+^ B cell levels were similar in patients with untreated RA and in controls. However, when RA patients were analyzed according to DAS28, we observed a moderate but significant negative correlation between the frequency of CD24^hi^CD38^−^ B cells from untreated RA patients and DAS28 (see Figure 2B).

When changes in the frequency of B cell subsets were evaluated by treatment, it was observed that the reduction in the number of CD19^+^CD24^hi^CD38^hi^ B cells in active RA patients is not conditioned by differential treatment regime (14). Interestingly, we observed that patients under treatment with anti-TNF or Tofacitinib exhibited a strong reduction in the frequency of CD19^+^CD24^hi^CD38^hi^ B cells (see Figure 1) and this reduction was not clearly associated with the disease status of the patients. Based on this observation we hypothesize that TNF and JAK-STAT pathways could be involved in the development/survival of the CD24^hi^CD38^hi^ B cell subset, because when these pathways were inhibited that population decreases. When all patients, treated and untreated, were analyzed, we found no correlation between B cell subset frequencies and DAS28 (Figure 2A), but we determined changes in B cell subsets in each individual linked to his/her successful response to therapy (changes in DAS28 values after 3 months of treatment).

The clinical significance and pathogenic roles of autoantibodies in RA are largely unknown except for RF and anti-citrullinated protein antibodies, whose clinical usefulness has been acknowledged due to their diagnostic sensitivity and specificity, and their prognostic value (42). Taking into account that the product of B cell subsets differentiation, the plasmablasts and plasma cells, are responsible for autoantibody production, we compared the changes in B cell subset frequency in RA patients, which were positive for RF (2). After treatment, CD24^hi^CD38^−^ B cells increased while CD24^hi^CD38^hi^ B cells decreased in R patients, and these changes were associated with a significant decrease in RF levels. Salomon et al. (38) reported an association between the presence of RF and lower absolute numbers of CD24^hi^CD38^hi^ B cells. Daien et al. (12) also observed that the levels of RF were inversely correlated with IL-10-producing B cells. A negative correlation between CD24^hi^CD38^hi^ B cells and RF and anti-citrullinated protein antibodies was also reported by Flores-Borja et al. (14), suggesting a “protective” role for regulatory B cells. CD24^hi^CD38^hi^ B cells in patients with pemphigus are elevated but with a defective regulatory function on Th1 cells (43), and thus it is important to consider not only the frequency and number of this population but also its functional activity. Our results indicate that CD24^hi^CD38^−^ B cells could exert a regulatory function on T cells and/or on autoantibody-producing B cells since their frequency increases after a positive response to treatment. Further studies are needed to confirm this hypothesis.

Available data (27, 41, 44) reinforce the importance of having an adequate number of Bregs to maintain the balance in the immune response. Here we find that B cells from RA patients can use mechanisms additional to IL-10 to control T cell response, such as the PD-1/PD-L1 pathway. All human peripheral blood B cell subsets expressed similar levels of the inhibitory molecule PD-L1, and the percentages of total CD19^+^PD-L1^+^ B cells as well as CD24^hi^CD38^−^PD-L1^+^ and CD24^hi^CD38^hi^PD-L1^+^ B cell subsets were lower in untreated RA patients than in HC. Notably, although the sample size of our study is really not large enough to obtain definitive conclusions, we observed that the frequency of CD19^+^, CD24^hi^CD38^−^ and CD24^hi^CD38^hi^ B cells that expressed PD-L1 increased in RA patients with a positive response to treatment. PD-L1, expressed on hematopoietic cells, binds to PD-1 to inhibit T cell receptor-mediated proliferation and induces T cell anergy (45). Guan et al. (46) reported that high PD-L1 expression may contribute to the immunosuppressive role of CD24^+^CD38^+^CD19^+^ Bregs in invasive breast cancer and that PD-L1^+^CD24^+^CD38^+^CD19^+^ Bregs may serve as a target in immunotherapy. Khan et al. (17) determined that PD-L1^hi^ B cells utilized PD-L1 as their main suppressive mechanism and that IL-10 was not required for PD-L1^hi^ B-cell suppressive activity.

Here, we report for the first time that PD-L1^+^ B cells from RA patients control CD8^+^ T cell proliferation and cytokine-production via PD-1/PD-L1 *in vitro*. Although B cells from HC and RA patients influenced CD4^+^ T cell response, they probably use another pathway in addition to PD-1/PD-L1 as, interestingly, blockade of the PD-1/PD-L1 interaction was not sufficient to reverse CD4^+^ T cell suppression. Studies in individuals with autoimmune diseases suggest that the PD-1/PD-L1 signaling pathway may exert an important regulatory function in Type I diabetes, LES, Sjögren's syndrome and also in RA (47), underlining the important role of PD-1 for maintaining self-tolerance.

In conclusion, our findings demonstrate a regulatory role for PD-L1^+^ B cells in RA patients *in vitro* and suggest that the PD-L1/PD-1 pathway may have a potential role in controlling disease activity since an increase in the frequency of these cells was associated with a positive response to treatment. PD-L1^+^ B cells from RA patients could thus provide new perspectives for future treatment strategies. Additionally, the phenotyping of B cells could have potential value for the stratification of patients in treatment response.

## Ethics statement

This study was carried out in accordance with the recommendations of Registro Provincial de Investigación en Salud (RePIS), Hospital Nacional de Clínicas ethical committee (CIEIS). The protocol was approved by the Hospital Nacional de Clínicas ethical committee (CIEIS). All subjects gave written informed consent in accordance with the Declaration of Helsinki.

## Author contributions

EZ performed and designed most of the experiments, analyzed data and prepared figures and manuscript. LIO, CA, and PF collaborated with the acquisition and interpretation of data. CM, EA and MR contributed to the study design and analysis and corrected the manuscript. SA contributed to biochemical determinations. EM, LO, IC, MS, and MT contributed to the recruitment and clinical evaluation of the patients. AG conceived, designed, and supervised the study and wrote the manuscript.

### Conflict of interest statement

The authors declare that the research was conducted in the absence of any commercial or financial relationships that could be construed as a potential conflict of interest.
